# Anthropometric Characteristics, Body Composition and Somatotype of Elite Male Young Runners

**DOI:** 10.3390/ijerph17020674

**Published:** 2020-01-20

**Authors:** Cristóbal Sánchez Muñoz, José J. Muros, Óscar López Belmonte, Mikel Zabala

**Affiliations:** 1 Department of Physical Education and Sport, University of Granada, 52071 Melilla, Spain; csm@ugr.es (C.S.M.); oscarlobel95@gmail.com (Ó.L.B.); 2 Department of Didactics of Musical, Plastic and Corporal Expression, University of Granada, 18071 Granada, Spain; 3 Department of Physical Education and Sport, University of Granada, 18071 Granada, Spain; mikelz@ugr.es

**Keywords:** anthropometry, body composition, somatotype, runners, youth

## Abstract

The purpose of the present study was to describe the anthropometric characteristics, body composition and somatotype of elite male young runners (EYR), and to compare these variables according to the specialty in which they engaged (middle-distance vs. long-distance). This will enable an anthropometric profile chart to be established for them. Ninety EYR aged 17 to 23 years (18.4 ± 2.0) participated in the study. Athletes were divided into two groups according to the event in which they participated: middle-distance runners (MDR, n = 56) and long-distance runners (LDR, n = 34). Sixteen anthropometric variables were recorded for each participant: Weight, height, eight skinfolds, four girths, and two breadths. Body mass index (BMI), body composition and somatotype were calculated. Comparing MDR with the group of LDR, significant differences were found to exist for height, weight, relaxed upper arm girth, flexed and tensed upper arm girth, total upper arm area, upper arm muscle area, and thigh muscle area. No significant differences were observed in the other variables. MDR are taller, heavier and have larger girths than LDR. Coaches and sports scientists can use the data obtained to better control training, as well as for talent identification and athlete selection.

## 1. Introduction 

In the last few years, issues that influence optimal performance in running events have received considerable attention in the scientific literature. Variables which have been associated with running performance include physical characteristics, maximal aerobic power (VO_2max_) [1,2,3,4,5], body composition [5,6,7], thigh-length [6], lactate threshold [8], the energy cost of running [9,10,11,12], running economy [10,13,14] and stride length [15,16].

Several researchers have published the physical characteristics of different types of runners [7,17,18]. Many studies have shown the anthropometric characteristics, somatotype and body composition of elite male adult runners [19,20]. However, to our knowledge, only two studies [18,21] have described these aspects in young elite male runners (EYR), and few studies have reported data for both individual and the sum of skinfold values amongst runners [18,22,23,24]. 

A moderate relationship between BMI and marathon running performance has been reported by Dotan et al. [25]. Arrese and Ostáriz [26] also reported lower limb skinfold thicknesses in males to be directly related to running time over 1500 m and 10,000 m. With regard to body composition, Brandon and Boileau [27] have reported that a larger fat-free mass enables runners to be more efficient. In addition, Wilson et al. [28] studied the relationship between somatotype and physical performance in running events. 

The aims of the present study were (1) to describe the anthropometric characteristics, body composition and somatotype of elite young runners (EYR), (2) to compare these variables according to participation in middle- or long-distance events, and (3) to establish an anthropometric profile chart for EYR.

## 2. Materials and Methods 

### 2.1. Subjects 

Ninety EYR aged 17 to 23 years old (18.4 ± 2.0) took part in the present study. All participants were national and international elite male runners. All participants were medalists in their aged category at the Spanish Championships, with fifteen of them being classified in the top ten at the European and World Championships. Runners were classified into two groups according to the event in which they participated: middle-distance (MDR, n = 56) (800 m and 1500 m) and long-distance (LDR, n = 34) (3000 m, 3000 m steeplechase and 5000 m) running events. Prior to measurement, all runners aged over 18 years gave informed consent to participate in the study. Completed parental consent forms were obtained for runners who were younger than 18 years old prior to them participating in the present research. The same runner could have participated in a number of MDR events (800 m or 1500 m, or both), these events all belonging to the same group of events. The same can be seen with those who participated in LDR. Evaluations were conducted at different meetings organized by the Andalusian Athletics Federation over a number of years. For all runners, data collection took place during a single day. Performance results were noted from the individual records of each athlete during the period in which they attended various meetings of the Andalusian Athletic Federation. The study was approved by the Ethics Committee of the University of Granada (n = 883) and was carried out in compliance with the Declaration of Helsinki.

### 2.2. Anthropometric Data 

Anthropometric measurements were performed following standardised techniques adopted by the International Society for the Advancement of Kinanthropometry (ISAK) [29] in basal conditions. This means that circumstances were avoided that affect the thickness and compressibility of skinfold measurements, such as previous exercise, baths, sauna sessions or dehydration states. Participants were measured barefoot, shirtless and were wearing shorts. All measurements were taken by the same investigator who was a Level 2 ISAK anthropometrist. Technical measurement error was lower than 5% for skinfolds and lower than 1% for all other measurements. Sixteen anthropometric variables were measured for each subject. These were: weight; height; thickness of 8 skinfolds (biceps, triceps, subscapular, suprailiac, supraspinal, abdominal, thigh and calf), 4 girths (relaxed upper arm, flexed and tensed upper arm, thigh and maximum calf), and 2 breadths (humerus and femur). Height was measured on a stadiometer to the nearest 0.1 cm (GPM, Seritex, Inc., Carlstadt, New Jersey) and body mass was recorded on a portable scale to the nearest 0.1 kg (model 707, Seca Corporation, Columbia, Maryland). Skinfold thickness was measured using a caliper calibrated to the nearest 0.2 mm (Holtain Ltd, Crymych, UK) and girths measurements were performed using a flexible anthropometric steel tape (Holtain Ltd, Crymych, UK) to the nearest 0.1 cm. Skinfolds were measured three times and the median was used in analyses. The sum of 3 skinfolds (triceps, subscapular, and supraspinal), the sum of 6 skinfolds (sum of 3 skinfolds and suprailiac, abdominal and thigh) and the sum of 8 skinfolds (sum of 6 and biceps and medial calf) were also calculated. BMI was calculated as weight/height^2^, where body mass was expressed in kilograms (kg) and height in metres (m). Body density was estimated using the equations of Durnin and Womersley [30], Katch and McArdle [31], Sloan [32], Wilmore and Behnke [33], and Withers et al. [34]. Density was transformed to %BF using Siri’s equation [35]. Muscle mass (MM) was determined in kg using the methods of Lee et al. [36]. Somatotype was determined using the Heath-Carter anthropometric method [37]. Performance was determined according to the time obtained in the race event in which athletes participated.

### 2.3. Statistical Analyses 

Standard descriptive statistics such as mean and standard deviation were used to present participant characteristics for all variables. The nonparametric Mann-Whitney test was used to compare anthropometric data between MDR and LDR groups. Statistical significance was set at *p* < 0.05. A profile chart was developed according to norms derived from percentiles (5 = lowest; 95 = highest). All statistical analyses were performed using the Statistical Package for the Social Sciences (version 21.0; SPSS, Inc, Chicago, IL, USA). 

## 3. Results 

Table 1 and Table 2 show the characteristics of the study sample and the results from the statistical analysis for differences between MDR and LDR. Mean height and weight of the assessed participants were 174.8 ± 4.7 cm and 61.8 ± 5.8 kg, respectively, with MDR being significantly taller and heavier than LDR (*p* = 0.010 and *p* = 0.002, respectively). In addition, we did not find significant differences in BMI between MDR and LDR. In relation to all girth and breadth measures, MDR had significantly higher relaxed upper arm girths and flexed and tensed upper arm girths than LDR (*p* = 0.046; *p* = 0.033, respectively). No significant differences were observed between MDR and LDR for the remaining girth and breadth measurements, skinfolds sums or %BF. 

An anthropometric profile chart was developed to evaluate the physical characteristics of young male runners (Table 3). In this profile, we can derive an overall evaluation of the physical characteristics of participants. The chart is useful as a screening device though all interpretations must be made in consideration of the specific individual context.

**Figure 1:**
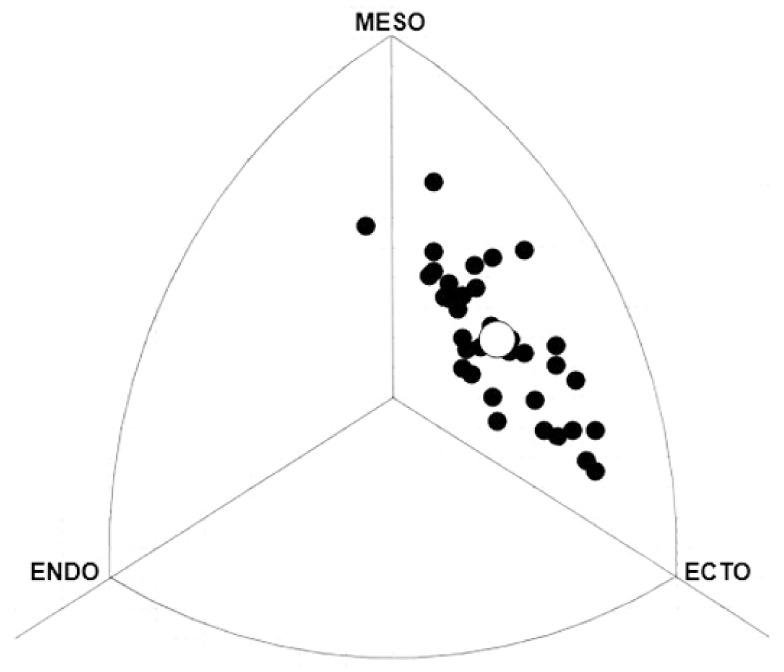
Somatotype distribution seen in young elite runners (n = 90). O = mean somatotype = 1.7-3.8-3.8 (endomorph, mesomorph, ectomorph).

The mean somatotype for evaluated EYR could be defined as ecto-mesomorphic (1.7-3.8-3.8). No significant differences were found in somatotype components between MDR and LDR (*p* < 0.05). Figure 1 presents a somatochart for young elite runners overall.

## 4. Discussion 

In agreement with Legaz Arrese et al. [18], the present results show that elite young MDR are taller and heavier than LDR. MDR also have larger girth and breadth dimensions. We were unable to find a similar study that compared the anthropometric characteristics of young elite male MDR and LDR. A comparison of the age, height, weight, BMI, %BF and performance of male junior [21,38,39] and senior [1,5,6,19,24,40,41,42,43,44] runners participating in different events is given in Table 4. The mean height of young male runners varies between 178 and 179 cm, with this being similar to that seen in senior MDR (177–178 cm). On the other hand, senior male MDR and LDR appear to be heavier than junior male runners. Our group of young male MDR is on average 2.6 cm smaller and 0.6 kg heavier than the young male runners evaluated in other studies. Further, our group of young male LDR is on average 4.2 cm smaller and 2.9 kg lighter than other examined young male runners.

In relation to skinfold thickness, our results indicated that no significant differences are present between MDR and LDR in the sum of skinfolds. In agreement with Legaz et al. [18], this result may be due to the fact that both groups of runners engage in a similar training volume. The skinfold values found in our runners are higher than values reported in other studies [18,45], except for the subscapular skinfolds of MDR which was lower. In comparison to Olympic runners [20], young runners involved in the present study had higher values for all skinfolds (except for subscapular skinfold) and the sum of six skinfolds. Only a handful of studies have reported data for individual skinfolds and the sum of values in relation to athletes of different ages [22,41,45]. Legaz et al. [18] found differences in the sum of six skinfolds between runners taking part in different events, but the present study did not find similar differences between MDR and LDR. The present study showed differences in upper arm girth, upper arm area, upper arm muscle area and thigh muscle area. The causes for such differences could be due to the type of training engaged in and subsequent nutrition strategy. Longer events typically need longer workouts and, therefore, a lower protein intake than events conducted over shorter distances. Performance over longer distances is also more dependent upon efficiency rather than efficacy, with lower muscle mass, especially in the upper limb, being key for this purpose. This makes sense as training is based on the quest for performance outcomes during competition. Longer distance events require athletes to be lighter and more efficient. This is achieved through a combination of their genetics, and training and nutritional strategies.

On the other hand, no significant differences were found in somatotype components between MDR and LDR. Carter [45] found that male Olympic runners were defined as ecto-mesomorphic (1.5-4.3-3.6 for MDR; 1.4-4.2-3.7 for LDR; and 1.4-4.4-3.4 for marathon runners), with no significant differences existing between Olympic runners taking part in different events. In accordance with previous studies [20], the mean somatotype of EMJR evaluated in the present study could also be defined as ecto-mesomorphic. When comparing the somatotype component values of young elite male MDR and LDR in the present study, with the somatotype component values of Olympic runners, young runners are seen to have somewhat higher endomorphy and ectomorphy values, and lower mesomorphy values.

All information pertaining to the anthropometrical characteristics, body composition and somatotype of Spanish elite junior runners, provides a frame of reference that can be used by coaches to better control the training process for the improvement of athlete performance and detect talent in running disciplines. All of these physical factors should be considered alongside performance, physiological, psychological and technical factors.

## 5. Conclusions

The present study provides reference values for anthropometric characteristics, body composition and somatotype of Spanish EYR in general, and MDR and LDR in particular. When comparing MDR with the group of LDR, significant differences were found to exist in height, weight, relaxed upper arm girth, flexed and tensed upper arm girth, total upper arm area, upper arm muscle area, and thigh muscle area. MDR could be defined as being more heavily built than LDR. Young Spanish elite male MDR are taller and heavier, with greater girth dimensions.

This study provides normative data that could help coaches with talent identification of young elite MDR and LDR.

Strengths: The main strength of the present study is the high quality of the studied participants. All were national and international elite male runners who were medalists in their age category at the Spain Championships. Fifteen of these were also classified in the top ten at the European and World Championships.

Limitations: Limitations of the present study include its cross-sectional design, which prevents conclusions from being made about the direction of associations. Results provide a frame of reference but should not be used as a fixed model for better performance. In this way, the results presented can be used as a standard reference but should be interpreted with caution in the context of individual characteristics and needs. Further, the chronological age of runners was considered instead of biological age. Thus, it is possible that some runners had not yet reached their maximum maturation levels and that differences exist in this aspect between runners.

## Figures and Tables

**Table 1:** Demographic characteristics of the study sample (mean ± SD) and differences between middle-distance runners and long-distance runners.

Dimension	Total Runners (N = 90)	Middle-Distance Runners (n = 56)	Long-Distance Runners (n = 34)
	Mean ± SD	Mean ± SD	Mean ± SD
Age (yr.)	18.4 ± 2.0	18.3 ± 2.1	18.3 ± 1.9
Height (cm)	174.8 ± 4.7	175.2 ± 4.7	174.1 ± 4.6
Weight (kg)	61.8 ± 5.6	62.4 ± 5.3	60.7 ± 5.9
BMI (kg/m^2^)	20.2 ± 1.5	20.3 ± 1.5	20.0 ± 1.6
Total years running (yr.)	6.2 ± 2.0	6.2 ± 2.1	6.1 ± 1.9
Training (hours/week)	12.2 ± 4.3	12.1 ± 4.4	12.5 ± 4.2
Performance (min:sec)	-	1:53.9 ± 4.2 ^a^ 3:53.6 ± 8.8 ^b^	8:22.2 ± 16.0 ^c^ 14:49.3 ± 23.8 ^d^ 9:12.1 ± 23.1 ^e^

*^a^* record 800 m; *^b^* record 1500 m; *^c^* record 3000 m; *^d^* record 5000 m; *^e^* record 3000 m steeplechase.

**Table 2:** Anthropometric characteristics, body composition and somatotype for young elite runners, (mean ± SD) and differences between middle-distance and long-distance runners.

Dimension	Total Runners (N = 90)	Middle-Distance Runners (n = 56)	Long-Distance Runners (n = 34)	*p*
	Mean ± SD	Mean ± SD	Mean ± SD	
**Skinfold thickness (mm)**				
Triceps (mm)	6.2 ± 1.4	6.2 ± 1.2	6.2 ± 1.6	NS
Biceps (mm)	3.0 ± 0.5	3.0 ± 0.5	3.1 ± 0.5	NS
Subscapular (mm)	7.1 ± 1.1	7.2 ± 1.0	7.0 ± 1.2	NS
Suprailiac (mm)	8.0 ± 2.2	7.9 ± 2.2	8.2 ± 2.2	NS
Supraspinal (mm)	4.9 ± 1.2	4.9 ± 1.3	5.0 ± 1.1	NS
Abdominal (mm)	7.6 ± 2.1	7.6 ± 1.9	7.7 ± 2.3	NS
Thigh (mm)	8.5 ± 2.3	8.2 ± 2.1	8.9 ± 2.6	NS
Calf (mm)	5.6 ± 1.3	5.5 ± 1.1	5.9 ± 1.5	NS
**Girth (cm)**				
Upper arm girth (cm) ^a^	25.6 ± 1.5	25.8 ± 1.5	25.2 ± 1.5	0.046 *
Upper arm girth (cm) ^b^	28.3 ± 1.5	28.5 ± 1.4	27.8 ± 1.5	0.033 *
Thigh girth (cm)	48.8 ± 2.9	49.3 ± 2.9	48.1 ± 2.8	NS
Calf girth (maximum) (cm)	35.6 ± 1.8	35.7 ± 1.9	35.4 ± 1.7	NS
**Breadth (cm)**				
Humerus (cm)	6.5 ± 0.3	6.5 ± 0.3	6.5 ± 0.4	NS
Femur (cm)	9.5 ± 0.4	9.5 ± 0.4	9.5 ± 0.4	NS
**Sum of skinfolds (mm)**				
Sum of 3 skinfolds (mm)	21.4 ± 3.7	21.3 ± 3.5	21.5 ± 4.0	NS
Sum of 6 skinfolds (mm)	45.5 ± 8.4	45.0 ± 8.4	46.4 ± 9.4	NS
Sum of 8 skinfolds (mm)	54.2 ± 9.7	53.5 ± 9.3	55.3 ± 10.5	NS
Sum upper limb skinfolds (mm)	37.0 ± 6.7	36.8 ± 6.5	37.2 ± 7.1	NS
Sum lower limb skinfolds (mm)	14.1 ± 3.3	13.7 ± 2.9	14.8 ± 3.8	NS
**Body fat (%)**				
Durnin and Womersley [30]	11.0 ± 2.3	11.0 ± 2.2	11.1 ± 2.5	NS
Katch and McArdle [31]	7.5 ± 1.1	7.5 ± 1.0	7.5 ± 1.2	NS
Sloan [32]	6.8 ± 1.6	6.7 ± 1.4	7.0 ± 1.8	NS
Wilmore and Behnke [33]	10.4 ± 1.0	10.3 ± 0.9	10.4 ± 1.1	NS
Withers et al. [34]	7.7 ± 1.2	7.6 ± 1.1	7.8 ± 1.3	NS
**Skeletal muscle mass (kg)** [36]	48.2 ± 2.3	48.3 ± 2.0	48.0 ± 2.8	NS
**Area (cm^2^)**				
Total upper arm area (cm^2^)	52.2 ± 6.2	53.2 ± 6.1	50.5 ± 6.0	0.046 *
Upper arm muscle area (cm^2^)	46.4 ± 5.6	47.4 ± 5.5	44.9 ± 5.6	0.038 *
Upper arm fat area (cm^2^)	5.8 ± 1.2	5.8 ± 1.1	5.7 ± 1.2	NS
Total thigh area (cm^2^)	190.5 ± 22.7	193.8 ± 23.0	185.0 ± 21.5	NS
Thigh muscle area (cm^2^)	170.4 ± 21.9	174.1 ± 21.3	164.2 ± 21.8	0.039
Thig fat area (cm^2^)	20.1 ± 5.5	19.7 ± 5.3	20.8 ± 5.9	NS
**Somatotype**				
Endomorphy	1.7 ± 0.4	1.7 ± 0.4	1.7 ± 0.4	NS
Mesomorphy	3.8 ± 0.9	3.8 ± 0.9	3.7 ± 0.9	NS
Ectomorphy	3.8 ± 0.9	3.8 ± 0.9	3.9 ± 0.9	NS

* *p* < 0.05; NS = non-significant; ^a^ Relaxed; ^b^ Flexed and tensed.

**Table 3:** Anthropometric profile chart for young elite runners (N = 90).

Dimension	Percentiles						
	5	10	25	50	75	90	95
Height (cm)	167.5	168.9	172.0	174.6	178.0	181.0	182.7
Weight (kg)	51.2	54.9	58.1	62.0	64.7	70.5	72.0
BMI (kg/m^2^)	17.4	18.2	19.2	20.2	21.2	22.3	22.7
Triceps skinfold (mm)	4.1	4.5	5.4	6.2	7.1	8.0	8.5
Biceps skinfold (mm)	2.0	2.5	2.7	3.0	3.4	3.7	3.9
Subscapular skinfold (mm)	5.4	5.6	6.2	7.3	8.0	8.4	8.6
Suprailiac skinfold (mm)	5.0	5.4	6.4	7.7	9.3	11.3	12.4
Supraspinal skinfold (mm)	3.5	3.5	4.2	4.6	5.7	6.3	7.1
Abdominal skinfold (mm)	5.0	5.3	6.3	7.3	8.6	10.5	12.4
Thigh skinfold (mm)	5.0	5.4	7.0	8.0	9.7	11.6	13.7
Calf skinfold (mm)	3.8	4.0	4.9	5.5	6.3	7.3	7.8
Upper arm girth (cm) *^a^*	23.0	23.7	24.5	25.5	26.7	27.8	28.0
Upper arm girth (cm) *^b^*	25.3	26.5	27.2	28.3	29.4	30.2	30.5
Thigh girth (cm)	43.5	45.1	47.0	49.0	51.2	52.5	53.4
Calf girth (maximum) (cm)	32.3	33.1	34.3	35.7	37.1	38.0	38.6
Humerus breadth (cm)	6.0	6.1	6.3	6.6	6.8	7.0	7.0
Femur breadth (cm)	8.9	8.9	9.2	9.5	9.8	10.1	10.3

*^a^* Relaxed; *^b^* Flexed and tensed.

**Table 4:** Summary table of studies examining age, height, weight, BMI, %BF and performance of elite runners participating in different events (mean ± SD).

Study	n	Age (year)	Height (cm)	Weight (kg)	BMI (kg/m^2^)	BF (%)	Time (s)	Event
Billat et al. [38]	8	18.0 ± 1.0	179.0 ± 4.0	62.0 ± 7.0	-	9.0 ± 1.5 *^b^*	800 m: 112.5 ± 3.8 1500 m: 235.3 ± 6.7	MD
Housh et al. [21]	26	17.3 ± 0.8	178.2 ± 6.8	63.6 ± 6.3	-	-	-	MD
Kenney and Hodgson [39]	8 5	21.4 ± 1.0 22.0 ± 1.5	- -	64.5 ± 2.4 71.9 ± 1.0	- -	8.8 ± 0.8 9.2 ± 0.5	5000 m: 845.0 3000 m steepl.: 518.0	LD
Billat et al. [19]	5	33.4 ± 2.0	172.0 ± 2.0	60.2 ± 2.9	-	-	-	Marathon
Billat et al. [40]	13	26.5 ± 3.6	170.0 ± 4.0	53.8 ± 4.7	-	6.6 ± 1.1 *^b^*	-	LD
Brandon and Boileau [6]	56	26.6 ± 4.5	179.5 ± 6.5	71.1 ± 8.2	-	13.7 ± 4.6 *^a^*	800 m: 141.0 1500 m: 291.0 3000 m: 637.0	MD
Deason et al. [5]	11	30.0 ± 3.9	177.5 ± 7.0	71.6 ± 8.0	-	-	800 m: 132.6 ± 7.3	800 m
Legaz and Eston [41]	23 16 8	21.8 ± 3.3 22.9 ± 3.2 22.0 ± 3.2 27.5 ± 4.1 26.0 ± 4.2 30.5 ± 3.9	- - -	69.3 ± 4.9 65.7 ± 3.9 66.1 ± 3.7 60.7 ± 4.2 58.0 ± 5.2 59.9 ± 3.3	- - -	- - -	800 m: 109.7 1500 m: 223.3 3000 m steepl.: 518.8 5000 m: 810.7 10,000 m: 1737.1 Marathon: 7980.3	MD LD
Maldonado-Martin et al. [1]	17	28.0 ± 5.0	178.0 ± 7.0	64.3 ± 7.0	-	12.4 ± 1.8 *^c^*	1500: 226.0 ± 4.2 Marathon: 8638.0 ± 401.6	1500 m/Marathon
Oguri et al. [42]	11	61.4 ± 3.0	165.4 ± 3.5	59.4 ± 4.9	21.8 ± 1.8	-	-	LD
Pollock et al. [24]	20	-	177.0 ± 6.0	63.1 ± 4.8	-	8.8 ± 0.8	-	LD
Pollock et al. [43]	40–49	-	180.7	63.1	-	8.8 ± 0.8	-	LD
Vernillo et al. [44]	14	27.7 ± 3.75	171.2 ± 6.1	57.7 ± 4.0	-	8.87 ± 0.07 *^d^*	Marathon: 7636.0 ± 115.0	Marathon
Present study	90 56 34	18.4 ± 2.0 18.3 ± 2.1 18.3 ± 1.9	174.8 ± 4.7 175.2 ± 4.7 174.1 ± 4.6	61.8 ± 5.6 62.4 ± 5.3 60.7 ± 5.9	20.2 ± 1.5 20.3 ± 1.5 20.0 ± 1.6	11.0 ± 2.3 *^b^*/7.5 ± 1.1 *^e^* 6.8 ± 1.6 *^f^*/10.4 ± 1.0 *^g^* 7.7 ± 1.2 *^h^* 11.0 ± 2.2 *^b^*/7.5 ± 1.0 *^e^* 6.7 ± 1.4 *^f^*/10.3 ± 0.9 *^g^* 7.6 ± 1.1 *^h^* 11.1 ± 2.5 *^b^*/7.5 ± 1.2 *^e^* 7.0 ± 1.8 *^f^*/10.4 ± 1.1 *^g^* 7.8 ± 1.3 *^h^*	- 800 m: 113.9 ± 4.2 1500 m: 233.6 ± 8.8 3000 m: 502.2 ± 16.0 5000 m: 889.3 ± 23.8 3000 m steeplechase: 552.1 ± 23.1	MD/LD 800 m/1500 m 3000 m/5000 m 3000 m steeplechase

*^a^* Brozek et al. [46]; *^b^* Durnin and Womersley [30]; *^c^* Durnin and Ramahan [47]; *^d^* Jackson and Pollock [48]; *^e^* Katch and McArdle [31]; *^f^* Sloan [32]; *^g^* Wilmore and Behnke [33]; *^h^* Withers et al. [34]. MD = Middle-distance running; LD = Long-distance running; - = Data not available.
